# Complete Genome Sequences of Classical Swine Fever Virus Subgenotype 2.1 and 2.2 Strains Isolated from Vietnamese Pigs

**DOI:** 10.1128/MRA.01634-18

**Published:** 2019-05-30

**Authors:** Ki-Sun Kim, Van Phan Le, SeEun Choe, Ra Mi Cha, Jihye Shin, In-Soo Cho, Nguyen Phuc Hung, Pham Ngoc Thach, Vu Thi Thu Hang, Dong-Jun An

**Affiliations:** a Animal and Plant Quarantine Agency, Gimcheon, Gyeongbuk, Republic of Korea; b College of Veterinary Medicine, Vietnam National University of Agriculture (VNUA), Hanoi, Vietnam; c Research and Development Laboratory, Rural Technology Development JSC, Hung Yen, Vietnam; KU Leuven

## Abstract

Classical swine fever virus (CSFV) strains ND20 and HY78 were detected from infected pigs in the Xuan Truong-Nam Dinh and Hung Yen provinces in North Vietnam in 2014 and 2015, respectively. The most prevalent CSFV subgenotypes in Vietnam are 2.1 and 2.2, and these two complete genome sequences will help the CSFV prevention policy in Vietnam. In particular, subgenotype 2.2 (ND20 strain) has been reported less worldwide, so it is worth sharing information about this subgenotype.

## Abstract (precis)

Classical swine fever virus (CSFV) strains ND20 and HY78 were detected from infected pigs in the Xuan Truong-Nam Dinh and Hung Yen provinces in North Vietnam in 2014 and 2015, respectively. The most prevalent CSFV subgenotypes in Vietnam are 2.1 and 2.2, and these two complete genome sequences will help the CSFV prevention policy in Vietnam.

## ANNOUNCEMENT

Classical swine fever virus (CSFV) is an enveloped virus containing a single-stranded positive-sense RNA genome of approximately 12.3 kb. The virus belongs to the genus Pestivirus within the family Flaviviridae (1). The virus causes a highly contagious disease in pigs, which results in significant economic losses to the pig industry both in Vietnam and worldwide. CSFV is categorized into three genotypes (1, 2, and 3), each comprising three to four subgenotypes (1.1 to 1.4, 2.1 to 2.3, and 3.1 to 3.4) (2, 3). Genetic analysis of the 5′ nontranslated region (NTR) of CSFVs isolated in the Mekong Delta area of South Vietnam between 2001 and 2003 shows that the strains clustered into two subgenotypes; the majority clustered into subgenotype 2.1 and the remainder into subgenotype 2.2 (4). In this study, we report the complete genome sequences of CSFV strains ND20 and HY78 detected from pigs with clinical symptoms of CSFV in 2014 and 2015, respectively. These pigs were from two pig farms in North Vietnam. Total RNA was extracted from blood samples using the RNeasy minikit (Qiagen, USA), and cDNA was synthesized with the HelixCript kit (NanoHelix, South Korea) using random hexamer oligonucleotides. Reverse transcription-PCR (RT-PCR) was conducted based on 6 previously reported primer sets (5). We designed 9 additional primer sets to properly cover the entire genome (available upon request). All PCR products were purified and cloned into vector pGEM-T, and the cloned genes were sequenced with an ABI Prism 3730xi DNA sequencer. To confirm the 5′ and 3′ end gene sequences, we also performed 5′/3′ rapid amplification of cDNA ends (RACE) PCR using the SMARTer RACE 5′/3′ kit (catalog number 634859; Clontech Laboratories, Inc.) following the manufacturer’s instructions, and PCR products were cloned into a pRACE vector and analyzed. The generated sequences were assembled and aligned using the BioEdit program, and the phylogenetic tree was analyzed using the Molecular Evolutionary Genetics Analysis (MEGA) 6.06 program (6).

**FIG 1:**
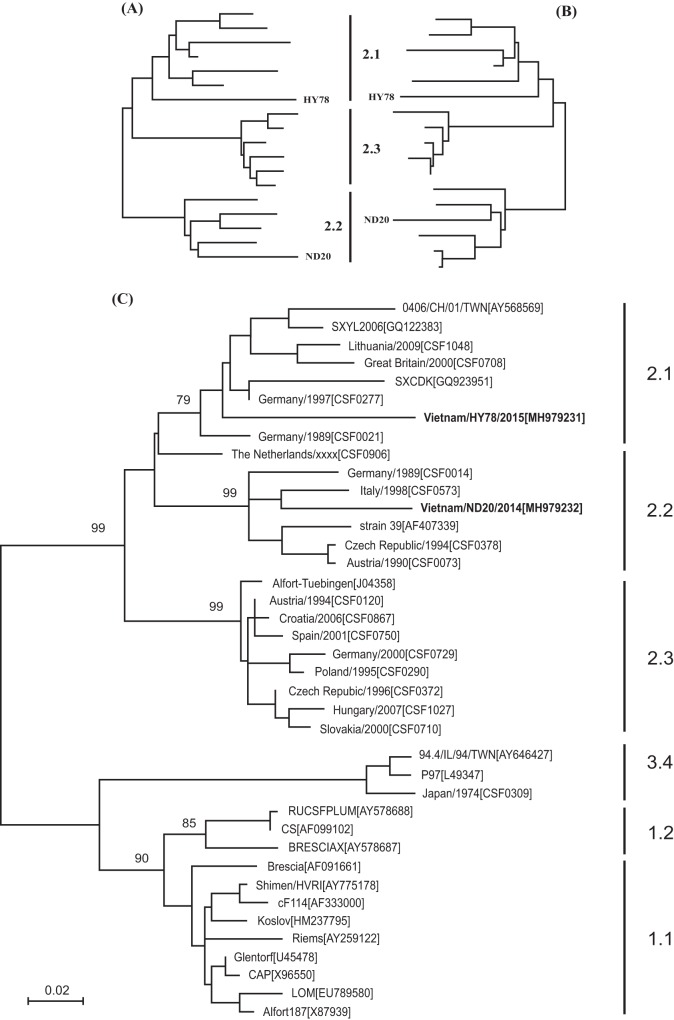
Phylogenetic trees based on the 5′-NTR fragment (A), the NS4B fragment (B), and the full-length E2 encoding sequences (C). Phylogenetic trees of 2 CSFV sequences detected from Vietnam and an additional 37 reference sequences originating from GenBank were calculated by the neighbor-joining method, including bootstrap values for 1,000 repetitions. Bootstrap values of >70% are shown at the breach points. The scale bar indicates the number of nucleotide substitutions per site.

The complete genome sequences of ND20 and HY78 were each 12,301 nucleotides (nt) long, including a 372-nt 5′ NTR, an 11,696-nt open reading frame (ORF) encoding a 3,898-amino-acid-long polyprotein, and a 231-nt 3′ NTR. The comparative gene analysis between ND20 (subgenotype 2.2) and HY78 (subgenotype 2.1) revealed sequence identities of 86.7% for the Npro genes, 82.1% for the C genes, 87.9% for the Erns genes, 88.3% for the E1 genes, 86.1% for the E2 genes, 87.1% for the P7 genes, 88.7% for the NS3 genes, 88.5% for the NS4A genes, 88.9% for the NS4B genes, 86.1% for the NS5A genes, and 88.3% for the NS5B genes. Based on the 5′ NTR, E2, and NS4B gene sequences, three neighbor-joining (NJ) trees were constructed using the MEGA 6.06 program, using ND20 and HY78 together with appropriate reference strains. ND20 and HY78 clustered together with subgenotype 2.2 and subgenotype 2.1 strains, respectively (Fig. 1). When complete CSFV genome sequences were compared with GenBank counterparts, strain HY78 showed 97.9% nucleotide similarity with strain GD53/2011 (GenBank accession number KP343640, subgenotype 2.1), which was isolated in China. In addition, the nucleotide sequence identity of strains ND20 and Nghe/2013, isolated in Vietnam, was 93.1%. The complete genome sequence of strain ND20 (subgenotype 2.2), derived from Vietnamese pigs, will provide valuable information to researchers.

### Data availability.

The complete genome sequences of the HY78 and ND20 strains have been deposited in GenBank under the accession numbers MH979231 and MH979232, respectively.
